# Blast-induced temporal alterations in blood–brain barrier properties in a rodent model

**DOI:** 10.1038/s41598-021-84730-8

**Published:** 2021-03-15

**Authors:** Usmah Kawoos, Rania Abutarboush, Ming Gu, Ye Chen, Jonathan K. Statz, Samantha Y. Goodrich, Stephen T. Ahlers

**Affiliations:** 1 Neurotrauma Department, Naval Medical Research Center, 503 Robert Grant Ave, Silver Spring, MD 20910 USA; 2 The Henry M. Jackson Foundation for the Advancement of Military Medicine Inc, Bethesda, MD USA

**Keywords:** Blood-brain barrier, Molecular neuroscience

## Abstract

The consequences of blast-induced traumatic brain injury (bTBI) on the blood–brain barrier (BBB) and components of the neurovascular unit are an area of active research. In this study we assessed the time course of BBB integrity in anesthetized rats exposed to a single blast overpressure of 130 kPa (18.9 PSI). BBB permeability was measured in vivo via intravital microscopy by imaging extravasation of fluorescently labeled tracers (40 kDa and 70 kDa molecular weight) through the pial microvasculature into brain parenchyma at 2–3 h, 1, 3, 14, or 28 days after the blast exposure. BBB structural changes were assessed by immunostaining and molecular assays. At 2–3 h and 1 day after blast exposure, significant increases in the extravasation of the 40 kDa but not the 70 kDa tracers were observed, along with differential reductions in the expression of tight junction proteins (occludin, claudin-5, zona occluden-1) and increase in the levels of the astrocytic water channel protein, AQP-4, and matrix metalloprotease, MMP-9. Nearly all of these measures were normalized by day 3 and maintained up to 28 days post exposure. These data demonstrate that blast-induced changes in BBB permeability are closely coupled to structural and functional components of the BBB.

## Introduction

Blast injuries are a significant contributor of chronic morbidities experienced by military service members. Between 2001 and 2018, more than 300,000 service members received at least one diagnosis of traumatic brain injury (TBI)^1^. In a study of a relatively small cohort of active duty military personnel with TBI, blast-induced traumatic brain injury (bTBI) was sustained by approximately one-third (36.3%) of the injured population^2,3^. The pathological mechanisms resulting from the propagation of a supersonic blast wave through the brain remain to be fully understood as does the association between blast exposure and the long-term neurological outcomes^4^. Poor neurological outcomes may be observed chronically in absence of overt pathology, such as bleeding, hematoma, and hemorrhage^5–8^. The underlying pathological mechanisms leading to clinical manifestations of bTBI may involve cerebral vascular changes.

Recent studies in rodents have shown acute and chronic cerebral vascular pathology indicating a vulnerability of the vasculature, including the integrity blood–brain barrier (BBB), to damage in response to blast exposure^9–12^. Increasing evidence suggests that exposure to blast elicits alteration of the BBB^6,13–21^. Exposure to blast was shown to disrupt the architecture and function of tight junctions (TJ) by modification or loss of trans-membrane (occludin and claudin-5) and cytoplasmic (zona occludens (ZO)—1–3) TJ proteins; and modification of junctional adhesion proteins (vascular endothelial (VE)—cadherins)^11,14,17–19,22^. A single blast exposure in the range of 60 kPa (8.7 PSI) to 180 kPa (26.1 PSI) was associated with a reduction in TJ proteins acutely at 0.25^18^, 4^18^, and 24 h^6^ post-blast with subsequent recovery in some cases at 24 h^18^. Blast-induced aberration in the regional distribution of claudin-5 was observed in the acute phase (1 h) after exposure to a single 106 kPa (15.4 PSI) BOP with recovery at 24 h^13^. However, three exposures, 24 h apart, at the same intensity resulted in relatively prolonged (24 h) disturbance in the distribution of claudin-5. A similar study examined the time course (up to 72 h) of region-specific effect of a single or double blast exposure on the distribution of claudin-5. Two 142 kPa (20.56 PSI) blast exposures within 10–12 min led to a significant decrease in claudin-5 in hippocampal CA1 region 72 h post-blast; no significant change in hippocampal claudin-5 density in the acute phase; and no effect on its distribution in dorsal straitum^19^. Additionally, the altered morphology of TJs was co-localized with micro domains of breach in the vascular permeability to blood-borne tracers and perivascular reactive gliosis.

Blast exposure has been shown to provoke increase in the permeability of BBB resulting in extravasation of the vascular components including serum proteins into the brain parenchyma^16,23,24^. Increased IgG reactivity in the brain was observed 3 and 24 h after an exposure to 126 kPa (18.3 PSI) BOP^23^ and 24 h after a single blast exposure to intensities varying from 130 (18.9 PSI) to 290 kPa (42 PSI)^25^. Blast injury-related increase in microvascular permeability to blood-borne tracers (such as sodium fluorescein, Evans blue, fluorescently labeled dextrans, and radiolabeled tracers) was previously reported with single or repetitive blast exposures^13,16–19^. Hue et al. characterized the time dependent (up to 24 h) and size-specific (376 Da–500 kDa) opening of the BBB after bTBI in rodents^15^, where the opening in the acute phase was large enough in size to allow tracers of less than 70 kDa to pass through the barrier followed by recovery of BBB integrity by 24 h after blast.

BBB disruptions in response to blast injury appear to occur transiently and are dependent on the magnitude of blast exposure(s), magnitude defined as the combination of blast intensity, repetitions, and the intervals between them^25^. Most studies that cover a wide range of blast exposure magnitudes have shown recovery of BBB properties by 10 days post-blast^15,17,19,24–27^. TBIs (including bTBI) have shown biphasic pattern of BBB opening, where the barrier re-establishes itself after the acute phase and reopens at a later point in time^19,28–32^. A single blast exposure was associated with increased BBB permeability to radiolabeled small (sucrose) and large (albumin) molecules in the acute phase with subsequent recovery by 3 days post-blast^19^. However, repetitive blast exposures at the same intensity evoked prolonged permeability to sucrose and a biphasic response to albumin within the same time frame. Blast-related BBB disruption studies to date have not assessed the longer-term repercussions of bTBI on the BBB that may possibly lead to a delayed opening of the barrier. Impairment in the morphology and function of the BBB during acute phases after the insult can trigger a cascade of secondary processes leading to vasogenic edema and neuroinflammation^33^. Primary subtle aberration in the acute phase after injury can also increase vulnerability of the BBB to damage by systemic inflammation especially in bTBI cases, where blast exposure is a whole body experience^34^.

Management of bTBI warrants a better understanding of the long-term alterations in the BBB and sequelae of blast-induced damage on the neurovascular unit on the whole. The study presented here focused on investigating time-dependent, blast-related alterations in the permeability of BBB with specific focus on functional properties and structural components of the barrier. The functional properties were investigated in vivo via intravital microscopy (IVM) and the structural aspect was assessed using immunostaining and molecular detection techniques.

## Results

This study assessed acute and chronic modifications to the permeability of the BBB and changes to associated components of the neurovascular unit as a result of a single exposure to blast. The effect of time lapse on BBB permeability after exposure to sham conditions was not significantly different among sham groups at 2–3 h, 1, and 3 days (D) post-sham treatment, as determined by IVM study (ANOVA: *F* = 0.709; adjusted *p* = 0.480 and 0.625 in comparison with 2–3 h for 1 D and 3 D, respectively). Similarly, Western blot analyses of TJ proteins (claudin-5 and occludin; claudin-5, ANOVA: *F* = 6.566, adjusted *p* = 0.12, 0.497, 0.186, 0.421 in comparison with 2–3 h for 1, 3, 14, and 28 D, respectively; occludin, ANOVA: *F* = 1.799, adjusted *p* = 0.997, 0.949, 0.923, 0.277 in comparison with 2–3 h for 1, 3, 14, and 28 D, respectively) at the specified time points post-sham treatment (2–3 h, 1, 3, 14, and 28 D) showed no significant change over time. Therefore, blast exposed group at each time point was compared with the sham group at 2–3 h post-sham treatment.

### Exposure to blast leads to an increase in size-specific BBB permeability

**Figure 1:**
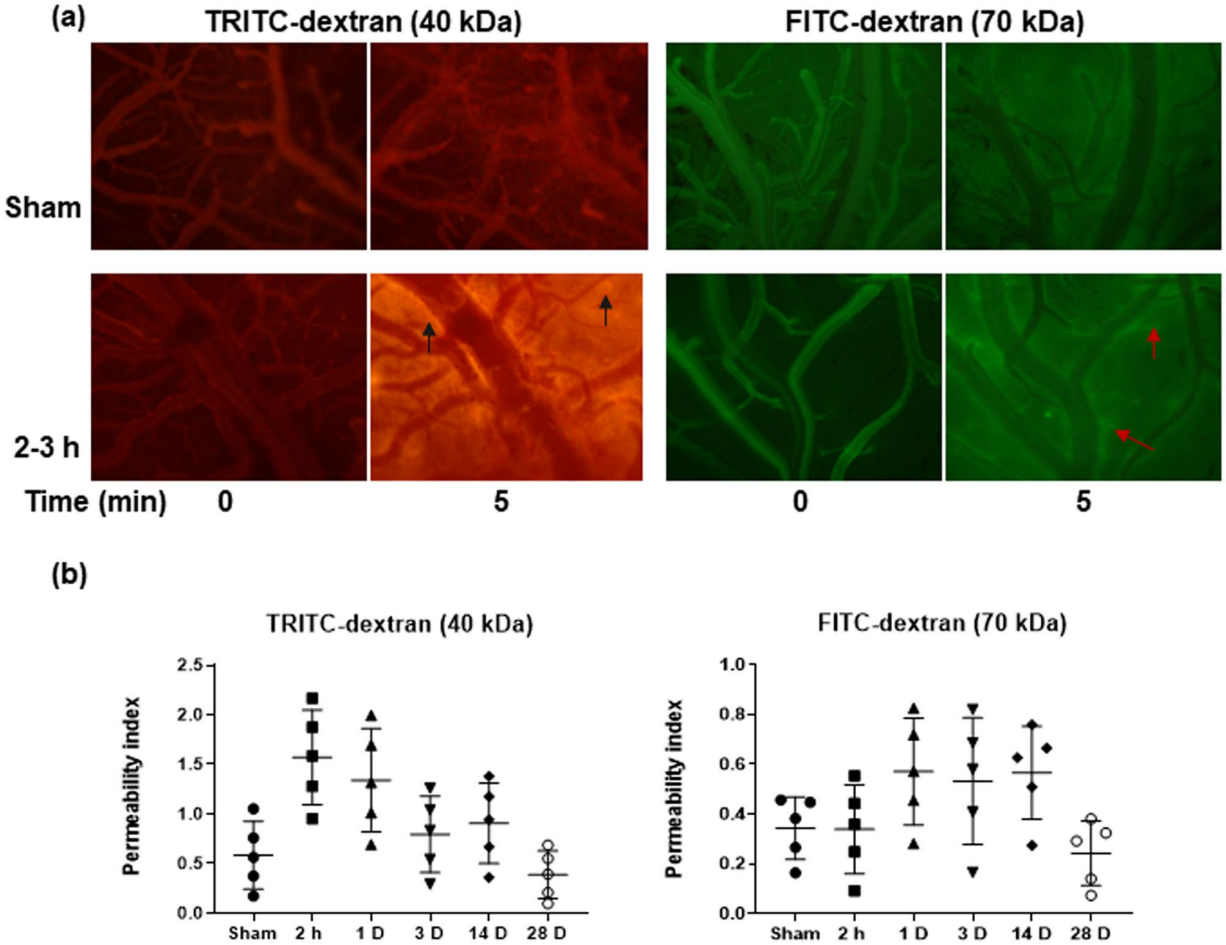
Extravasation of fluorescent tracers TRITC-and FITC-dextran from pial microvessels. (**a**) Representative intravital microscopy images of TRITC- and FITC-dextran in pial microcirculation and their extravasation in the brain parenchyma immediately (0 min) and 5 min (5 min) after the administration of fluorescent tracers in sham and 2-3 h groups. Arrows indicate sites of dextran in the extravascular space, and (**b**) Permeability index representing the degree of extravasation of TRITC- and FITC-dextrans through the vasculature. Data are mean ± SD.

The integrity of BBB was compromised in the acute phase (up to 1 D) after blast exposure in a size-specific manner, with the barrier permitting the passage of smaller tracer (40 kDa) molecules, while maintaining its relative impermeability towards the larger molecule (70 kDa). Figure 1a shows representative intravital images of the pial microvasculature under sham and blast (2–3 h) conditions with intense (red) and slight (green) extravasation of the smaller and larger tracer, respectively. As these data failed to meet the assumptions of normality, the data was transformed using the aligned rank transform^35^ such that a two-way repeated measures ANOVA (*F* = 1.96, adjusted *p* = 0.11, no-post hoc analyses were conducted) could be applied. In comparison to sham, an increase in PI (permeability index) of the 40 kDa tracer by approximately 2–3 times was observed post-blast at 2–3 h and 1 D, with slightly increased PIs at 3 and 14 D, and near sham values at 28 D (Fig. 1b). When comparing the values of PI for the tracers under sham conditions, the values were higher for the smaller of the two tracers but the differences in means for the two sizes were not statistically significant (two-way ANOVA: adjusted *p* = 0.171), which provides an indication of the barrier’s integrity in uncompromised state. A single blast exposure led to size-specific alteration of the BBB in the short-term after blast followed by natural recovery in the function (as seen by reduction in the passage of tracers through the BBB) of the barrier by 28 D post-blast.

### Exposure to blast reduces the expression of TJ proteins

**Figure 2:**
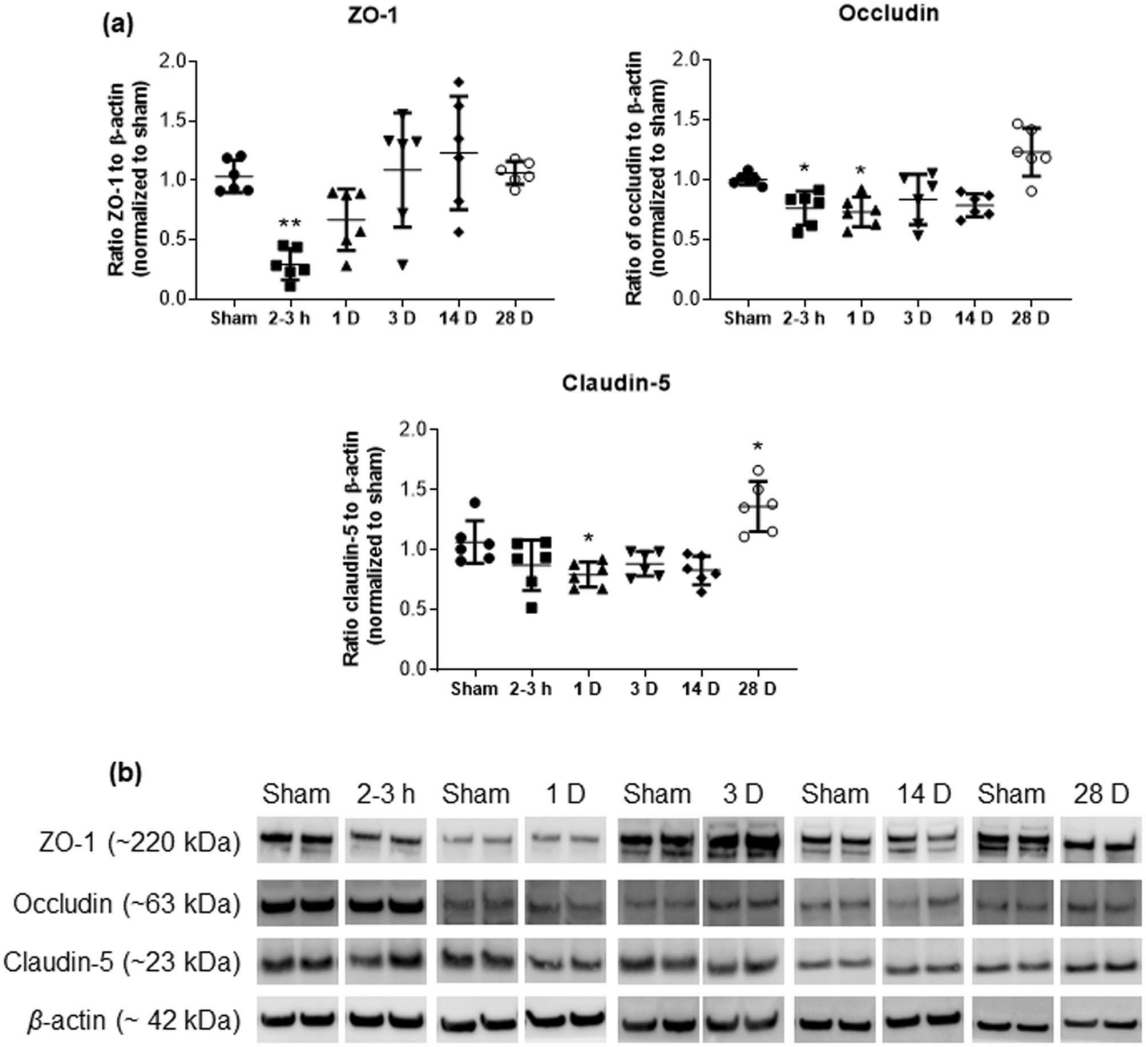
(**a**) Semi-quantitative assessment of Western blot band optical density showing blast-induced changes in tight junction (TJ) proteins over time. Exposure to BOP leads to decrease in the expression of TJ proteins, ZO-1 and occludin, 2-3 h post-blast and that of claudin-5 and occludin 1D post-blast. All three proteins exhibit an upward trend at ≥ 3D post-BOP exposure. (**b**) Representative Western blot bands for two animals per group. Complete Western blot bands are provided in supplementary file. Data are mean ± SD, *p < 0.05, **p < 0.01 for comparison of the specified time point post-blast with sham.

Breach of the vascular permeability barrier after exposure to blast and the associated extravasation of plasma constituents, i.e., the fluorescently-labeled dextrans in the IVM studies described here, coincided with decreases in the expressions of TJ proteins ZO-1, occludin, and claudin-5 that reached statistical significance 2–3 h and 1 D after blast exposure. In particular, western blot analyses showed a significant decrease in the levels of TJ proteins 2–3 h post-blast (Fig. 2), with ZO-1 exhibiting the greatest decrement and decreasing to less than a third of the ZO-1 levels in sham. The protein levels of occludin and claudin-5 remained significantly lower at 1 D post-blast than their levels in the sham group. Although not statistically significant, there was a persistent decrease in the protein levels of occludin and claudin-5 (but not ZO-1) after blast exposure that lasted up to 14 D. The levels of both proteins exhibited a perhaps compensatory increase above sham levels 28 D post-blast (post-hoc tests of sham versus 28 D claudin-5 were statistically significant). Statistical and descriptive values are presented in Table 1.

**Table 1:** Statistical and descriptive values of significantly different results.

Variable/protein	ANOVA/Kruskal–Wallis statistic	*p*	Effect size (*η*^2^)	95% Confidence interval	Group	Mean/median	SD( ±)/IQI	Dunnett’s/Dunn’s *p* (to Sham)
**Expression of TJ proteins—Western blots (Fig. ****2****)**								
ZO-1	*F* = 7.70	< 0.0001	0.56	0.22, 0.66	Sham	103.5%	13.7	
					2–3 h	29.4%	13.1	0.0011
Occludin	*F* = 10.06	< 0.0001	0.63	0.31, 0.71	Sham	100.3%	4.7	
					2–3 h	76.2%	14.1	0.0353
					1 D	73.0%	12.5	0.0143
Claudin-5	*F* = 10.67	< 0.0001	0.64	0.33, 0.72	Sham	106.6%	17.6	
					1 D	79.5%	10.4	0.0269
					28 D	136.1%	20.9	0.0141
**Expression of TJ proteins—immunostaining (Fig. ****3****)**								
ZO-1	*F* = 5.26	0.0021	0.52	0.11, 0.63	Sham	49.92 AU	20.03	
					2–3 h	29.17 AU	7.14	0.0203
					1 D	24.21 AU	7.74	0.0035
Occludin	*KW* = 18.23	0.0027			Sham	46.92 AU	44.68–47.62	
					2–3 h	27.94 AU	26.34–30.42	0.0143
Claudin-5	*KW* = 14.73	0.0116			Sham	63.30 AU	59.23–65.91	
					2–3 h	42.37 AU	39.75–48.52	0.0061
					1 D	40.06 AU	38.58–42.55	0.0061
**Expression of AQP-4 in cortex (Fig. ****4****)**								
AQP-4	*F* = 5.258	0.0021	0.52	0.11, 0.63	Sham	17.14 AU	2.87	
					2–3 h	21.68 AU	1.74	0.0439
					1 D	23.08 AU	2.12	0.0061
**Degradation of ECM, MMP-9 expression (Fig. ****5****)**								
mRNA	*F* = 5.487	0.0013	0.50	0.12, 0.62	Sham	1.02	0.21	
					2–3 h	2.67 fold	0.79	0.0115
					1 D	2.72 fold	1.32	0.0128
					3 D	3.19 fold	1.02	0.0013
Tissue	*F* = 7.164	0.0002	0.54	0.20, 0.65	Sham	2.45 ng/mg	0.76	
					2–3 h	3.63 ng/mg	0.58	0.0193
					1 D	4.26 ng/mg	0.98	0.0003
Plasma	*F* = 6.847	0.0003	0.54	0.18, 0.65	Sham	6.53 ng/ml	0.93	
					2–3 h	7.98 ng/ml	0.42	0.0007
					1 D	7.69 ng/ml	0.63	0.0068

*ANOVA* analysis of variance, *IQI* interquartile interval, *TJ* tight junction, *ECM* extracellular matrix.

**Figure 3:**
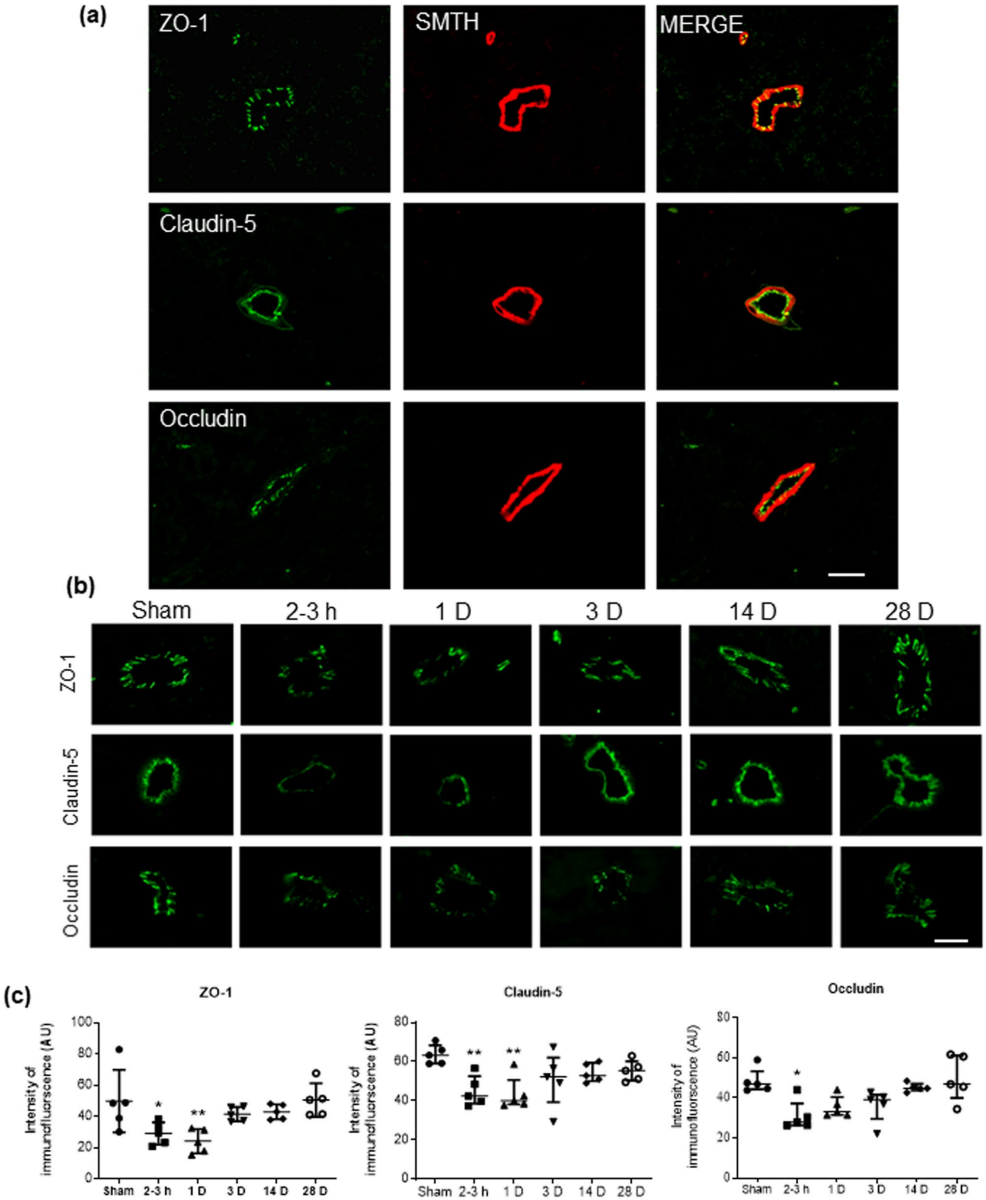
Decrease in the expression of tight junction (TJ) proteins in cortex was induced by BOP. (**a**) Co-localization of TJ proteins ZO-1, claudin-5, and occludin with SMTH, the blood vessel smooth muscle marker in sham rats. (**b**) Immunofluorescent images of TJ proteins in sham and blast-exposed rats at different time points post-BOP. (**c**) Immunofluorescence intensity assessment of TJ proteins in sham and blast-exposed rats. BOP induced significant reduction in the expression of TJ proteins at 2 h and 1D after exposure, followed by an increasing trend towards sham levels at later time points. Scale bar in (**a**) 25 μm. Data are mean ± SD, *p < 0.05, **p < 0.01 for comparison of the specified time point post-blast with sham.

Results from double-immunolabeling of junctional proteins with the vascular smooth muscle marker smoothelin (SMTH) revealed, expectedly, that these proteins were localized to the microvasculature in the cortex (Fig. 3a). Specifically, ZO-1 and occludin immunostaining was observed between cells co-localizing with SMTH, presumably endothelial cells. Claudin-5 immunoreactivity lined the luminal surface of the SMTH-immunostained smooth muscle cells. Examination of the time course of blast-induced changes in the expression of junctional proteins in the cortex using immunostaining and confocal microscopy also showed remarkable changes in immunofluorescence intensity of TJ proteins 2–3 h and 1 D after blast exposure (Fig. 3b). Semi-quantitative analysis of immunofluorescence intensity of ZO-1 (Fig. 3c) showed a significant decrease in intensity 2–3 h post-BOP compared to the sham group, and continued to decrease 1 D post-BOP, which was 52% lower than ZO-1 immunofluorescence in sham rats. Similarly, the immunofluorescence intensity of claudin-5 (Fig. 3c) and occludin (Fig. 3c) also decreased significantly post-BOP (sham versus 2–3 h and 1 D post-blast for claudin-5; sham versus 2–3 h post-blast for occludin). Immunofluorescence intensity for all three TJ proteins gradually increased beginning at 3 D post-BOP, reaching near sham levels at 28 D post-BOP. Statistical and descriptive values are presented in Table 1.

### Exposure to blast induces increase in the expression of AQP-4 in cortex

**Figure 4:**
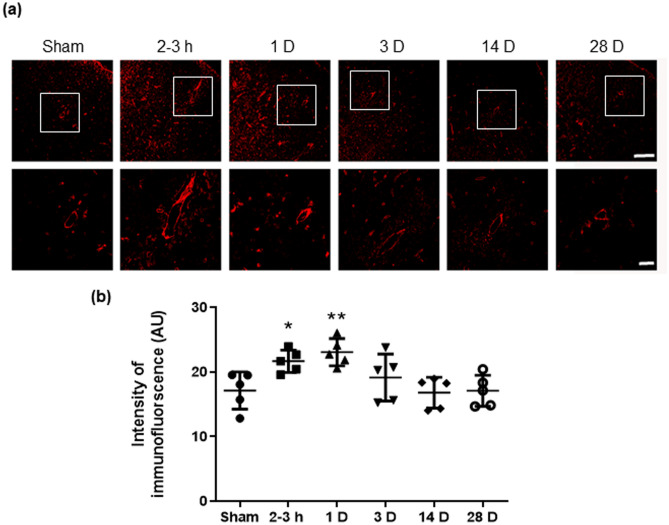
Increase in the expression of AQP-4 in cortex was induced by blast. (**a**) Representative immunofluorescent images of AQP-4 in sham and blast-exposed groups at different time points post-BOP. (**b**) Immunofluorescence intensity assessment of AQP-4 in sham and blast-exposed rats. BOP induced a significant increase in the expression of AQP-4 at 2-3 h and 1D after exposure, then returned to normal from 3D after exposure. Scale bar in (**a**) 100 μm and 25 μm in high magnification inserts. Data are mean ± SD, *p < 0.05, **p < 0.01 for comparison of the specified time point post-blast with sham.

The upregulation of AQP-4, expressed predominantly in astrocyte foot processes, has been shown to facilitate resolution of cerebral cytotoxic edema after TBI^36^. Compared with the sham group, the immunofluorescence intensity of AQP-4 in blast exposed groups was significantly increased as early as 2–3 h and continued to be higher at 1 D post-BOP as shown in Fig. 4. The expression of AQP-4 in blast-exposed groups returned to near sham levels at the subsequent time points after 1 D, this pattern of changes in AQP-4 being complementary to the expression of TJ proteins post-blast (Fig. 2). Statistical and descriptive values are presented in Table 1.

### Degradation of ECM after exposure to blast

**Figure 5:**
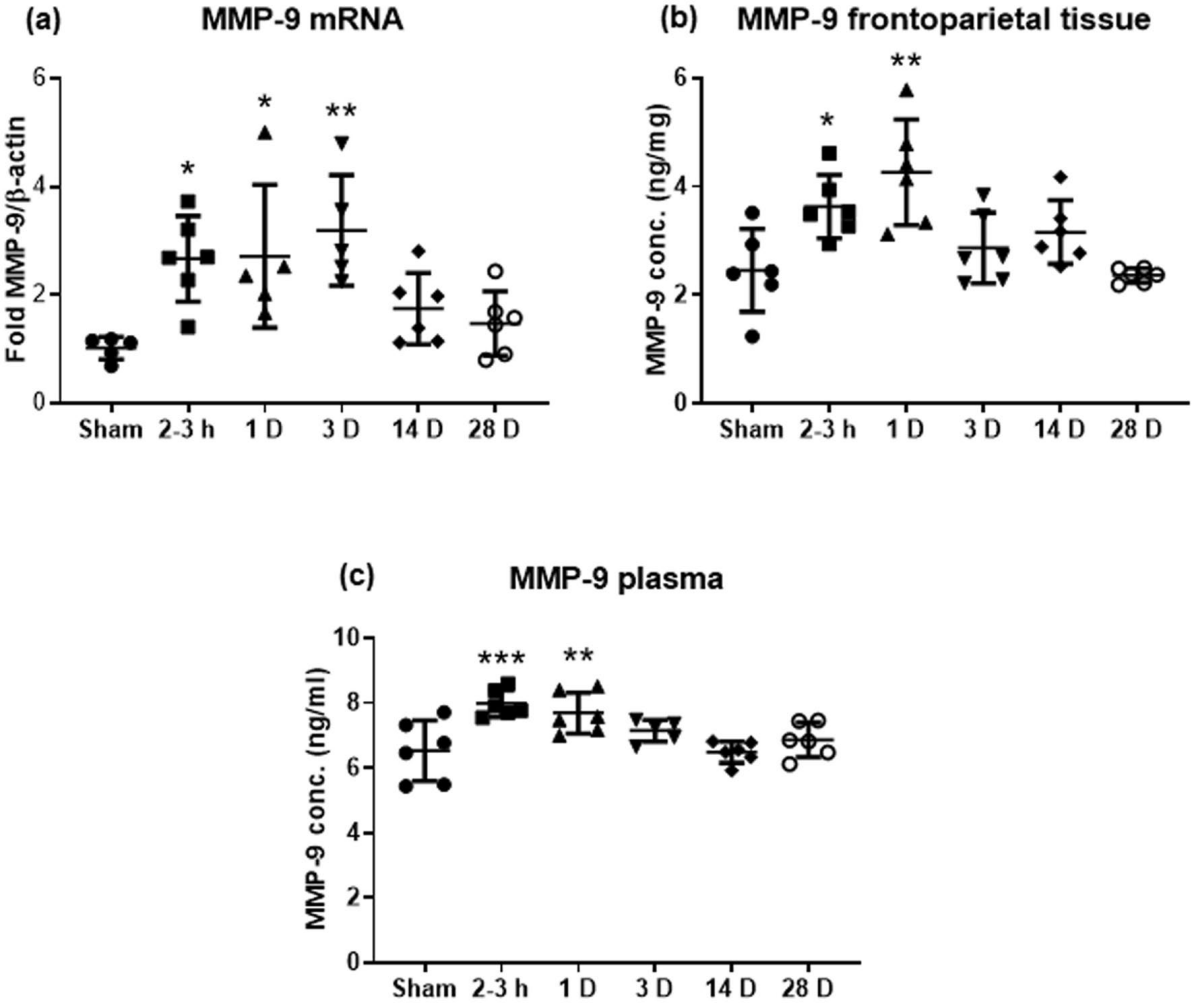
Up-regulation of MMP-9 in plasma and frontoparietal cortex was induced by blast. Changes in (**a**) MMP-9 mRNA, (**b**) MMP-9 protein, and (**c**) plasma MMP-9. Blast induced significant up-regulation of MMP-9 at 2-3 h and 1D after exposure. Data are mean ± SD, *p < 0.05, **p < 0.01, ***p < 0.001 for comparison of the specified time point post-blast with sham.

The changes in expression of MMP-9 were examined due to its involvement in the cleavage of ECM and the association between disruption of the ECM and BBB dysfunction in the microvasculature^37^. Under pathophysiological conditions, activation of MMP-9 is associated with opening of the BBB and degradation of TJs and the neurovascular unit basement membrane^38^. We observed a significant increase in MMP-9 mRNA in brain tissue at 2–3 h, continuing a significantly upward trend at 1 D and 3 D post-blast, where a ~ threefold increase in comparison to sham was seen (Fig. 5a). This increase in mRNA was paralleled by a significant increase in protein levels in both brain tissue and plasma 2–3 h and 1 D after blast exposure (Fig. 5b, c). For MMP-9 protein analyses, adjusted *p* values for Dunnett’s multiple comparison test in comparison to sham for tissue MMP-9 were significant for the 2–3 h and 1 D groups. For plasma MMP-9, the Dunnett’s multiple comparison test adjusted *p* values were also significant for the 2–3 h and 1 D groups compared to sham. Statistical and descriptive values are found in Table 1.

To summarize, the effect of a single 130 kPa (18.9 PSI) blast exposure led to time-dependent alterations of the BBB that were associated with modifications in the structural components or factors that modulate the integrity of the BBB. The increase in BBB permeability was accompanied by attenuated expression of the junctional and cytoplasmic TJ proteins (occludin, claudin-5, and ZO-1), up-regulation of astrocytic water channel protein (AQP-4), and activation of ECM cleaving MMP-9. The reversal (over time) of the aberrant functional property was also accompanied by restoration of the studied structural constituents of BBB.

## Discussion

The factors contributing to the maintenance of the BBB are sensitive to insults of various natures that include trauma to the brain (concussions, closed head injury, penetrating or blunt force trauma, bTBI)^18,19,22,24,39–42^, deprivation of adequate oxygenation (cerebral hemorrhage, ischemic stroke, hypoxia)^43–51^, infections (retroviral, meningitis, sepsis)^52–54^, and brain disorders^55–57^. Disruption in the function of the BBB, triggered by the primary insult, is caused by imbalance in the interplay of multiple complex and inter-related mechanisms. Our study showed that a single exposure to primary blast wave resulted in the onset of processes which altered the balance maintained in the neurovascular unit leading to disruption in the endothelial layer, astrocytic processes, and the ECM together with compromised integrity of the BBB. We performed a longitudinal assessment of the alterations and subsequent recovery over a period of 28 days following blast exposure. The size specificity in the disruption of BBB permeability as seen by increased extravasation of 40 kDa tracer and unremarkable leakage of 70 kDa in our study was also reported by others^13,15,19^. Hue et al.^15^ characterized the time-course and the size of BBB opening after exposing mice to 272 kPa (39.45 PSI) BOP and reported extravasation of tracers with molecular weights of less than 70 kDa within a few hours of injury, similar to our findings. However, in their model the permeability in BBB returned to near baseline state 24 h after blast, whereas our study with lower blast intensity (130 kPa, 18.9 PSI) showed a persistent increase in permeability of the smaller tracer for 24 h after BOP exposure. Logsdon et al.^19^ examined blast-induced BBB permeability changes to radiolabeled sucrose and albumin-tracers used to assess permeability of small and large blood-borne molecules, respectively. A single blast exposure at 141.8 kPa (20.56 PSI) was associated with a persistent increase in BBB permeability (for up to 72 h) of the smaller molecule and a significant increase in the permeability of the larger molecule only in the acute phase (0.25 h) post-blast. In other studies, exposure to 120 kPa (17.4 PSI)^24^ or 241 kPa (35 PSI)^58^ blast resulted in increased IgG immunoreactivity at 3 h^24^ and 24 h^24,58^ with recovery at 3 days post-blast^58^. The modest disparities in these findings are likely due to differences in the loading conditions of blast injury, which include placement of the animal (inside or outside the blast simulator), orientation of the animal with respect to BOP, overpressure characteristics, difference in species, and linear and rotational head acceleration.

In TBI models including controlled cortical impact, fluid percussion, acceleration-deceleration, and bTBI there is growing evidence of BBB breakdown in acute and delayed phases after injury^59–63^. The primary or acute stage of bTBI is a response to impulsive mechanical forces that results in shearing, contusions, and hematomas. Several studies have reported the association of TBI-induced aberrant BBB permeability in the acute phase with neurovascular inflammation, astrogliosis, mural (smooth muscle cells and pericytes) and glial cell dysfunction, and damaged vascular and paravascular ultrastructure^9,12,26,64^. Even though there are similarities in pathologies associated with bTBI and TBI caused by other mechanisms, bTBI presents distinct features^65–70^. The uniqueness of bTBI may be attributed to heterogeneity in the viscoelastic properties of brain structures, which are responsible for inhomogeneous stress–strain response, across brain regions, to the incident impulse of high energy (blast wave) leading to structural abnormalities like petechial hemorrhaging and axonal injury^39^. Since exposure to blast is typically a whole body experience, it leads to activation of interrelated systemic and cerebral mechanisms that are critical in defining the trajectory of blast-induced neurotrauma^71–73^.

The mechanical loading of blast on cerebral vasculature can result in micro-tears in blood vessels^70^ resulting in immediate alterations in the endothelial component of the BBB and consequently affecting the stability of TJs and their associated proteins. Our study showed a significant reduction in ZO-1 and occludin immediately (2–3 h) after blast exposure with occludin and claudin-5 remaining significantly decreased 1D post-blast and all three TJ proteins approaching sham levels at 3 D post-blast. The pattern of post-blast decrement in TJ proteins in cortex over time was paralleled by correlating dysfunction in the pial microvascular permeability of BBB as seen by increase in the extravasation of the 40 kDa tracer in vivo following blast exposure. Although there may be regional differences in the mechanical stress induced by blast on the cerebral vasculature, studies have shown extravasation of blood-borne tracers from cortical vessels in the acute phase (3 and 24 h) in a bTBI model that is similar to our study^15,24^.

In the sub-endothelial microenvironment, astrocytes and the ECM influence the homeostasis of the BBB. Astrocyte derived vascular permeability factors such as vascular endothelial growth factor (VEGF), nitric oxide (NO), endothelins, and MMPs are known to promote BBB permeability by modulating TJ complexes and increasing para-endothelial transport^41^. Disrupted BBB permeability leads to reactive astrogliosis and increase in the expression of AQP-4^74^. Astrogliosis is accompanied by increase in the expression of AQP-4 which plays a vital role in the formation and resolution of vasogenic edema^36^. Increase in m1 isoform of AQP-4 disrupts the sites of AQP-4 localization which inhibits movement of water, leading to an increase in hydrostatic pressure^36^. During the edema resolution phase, upregulation of AQP-4 is associated with an increase in water clearance from tissue followed by reductions in microglial activation, production of MMPs, disruption in the TJs and restoration of BBB properties. In our previous study, a single exposure to 110 kPa (15.95 PSI) BOP led to a persistent increase in the intracranial pressure (ICP) for up to 2 D post-blast followed by a gradual decrease (starting at 3 D) in the pressure^17^. Our present study showed significant blast-induced increase in the expression of AQP-4 in the acute phase (2–3 h and 1 D) followed by return to near sham levels at later time points. Taken together, the changes in AQP-4 and ICP are suggestive of the role of AQP-4 in disruptive and reparative processes following bTBI. Time-matched elevated levels of MMP-9 in plasma and frontoparietal cortical tissue paralleled the pattern of changes observed with AQP-4.

Under normal physiological conditions, MMPs have a key role in maintaining the dynamic integrity of the BBB and the neurovascular system by supporting the ECM. However, pathological conditions can trigger upregulation of MMPs causing disruption to the ECM, microvascular basement membrane proteins, and endothelial membrane proteins with exacerbated BBB permeability^75^. Brain injury was shown to trigger astrocytic activation of MMP-9 and compromise in the integrity of BBB; whereas inhibition of MMP-9 activation ameliorated TBI-induced damage to the barrier^76^. Studies with single or repetitive bTBI in rodents have shown increase in MMPs (− 2, 3 and 9) for up to 24 h after injury with a gradual reduction in the level of MMP-2 after 6 h of blast exposure^26^. Elevation in the levels of MMP-9 and astrogliosis marker, glial fibrillary acid protein (GFAP), was reported in human dissociated CNS cultures 24 h after exposure to blast-like shock waves^77^. Interestingly, post-blast levels of MMP-9 mRNA in tissue continued to be elevated for 3 days. One possible explanation is decline in the translation of the protein due to progressive reversal of vascular microenvironment’s pathological conditions to near baseline state by the third day after blast. This timeline of the reversal is supported by the pattern of changes observed in TJ proteins, AQP-4, and BBB permeability at 3 D post-blast at which time the barrier appears to be reestablishing itself. This is further corroborated by our previous findings that show a reduction in blast-induced elevated ICP starting at 3 days post-blast^17^. Longsdon et al. also reported BBB disruptive and reparative processes within 3 D after exposure to a single blast leading to size-specific changes in BBB permeability and partial restoration within this time frame^19^.

Brain endothelin-1 (ET-1) is a potent vasoactive neuropeptide that modulates vascular tone and BBB permeability. ET-1 levels are increased in a variety of pathological states including ischemia, subarachnoid hemorrhage, vasospasm, and TBI^78–81^. ET-1 is typically released by endothelial cells; however, under some pathological conditions, including TBI, reactive astrocytes express ET-1 and the endothelin receptors, specifically the ET_B_ receptor^82^. ET-1 overexpression has been shown to exacerbate BBB dysfunction and edema formation in stroke and impact TBI models^81,83^. Administration of ET_B_ receptor antagonists attenuated BBB disruption and edema in rodent TBI models^81^. The adverse effects of increased levels of ET-1 after CNS injury may be mediated by activation of MMP-9^84^ and induction of reactive astrocytes through the ET_B_ receptor. Another BBB permeability regulatory peptide found in smooth muscles and endothelium is myosin light chain (MLC) and enhanced phosphorylation of MLC (p-MLC) is associated with vascular pathologies including hyperpermeability of the BBB^85^. Increase in p-MLC can induce disorganization or degradation of TJ proteins leading to BBB disruption^86^. In the same bTBI model as examined in the current study, we have previously shown an increase in both the biologically active ET-1 and its precursor big ET-1 within 2 h of exposure to blast^87^. In the same study, we demonstrated a delayed 30% increase in the astrocytic ET_B_ receptor by 28 days post-blast. The study also showed a delayed increase in p-MLC levels by ~ 18% at 14 D and decrease by 75% at 28 D post-blast when compared to sham. The changes in p-MLC may be a contributing factor for alterations in TJ proteins, claudin-5 and occludin in the early phase followed by reversal to near-sham levels by 28 D post-blast. These data, together with the findings of the current study, demonstrate that the observed BBB breakdown, increase in MMP-9, and reduction of occludin, claudin-5 and ZO-1 within the first 24 h post-blast may be triggered by the early increase in ET-1, as shown in other neurological conditions. Although the source of the elevated ET-1 in our blast model remains to be determined, dysregulation of astrocytes may be an important contributing factor to increased ET-1 levels and altered BBB integrity. Mechanical stress has been shown to promote production of ET-1 by astrocytes^88,89^. Given the increase in AQP4 expression reported here and the elevation in ICP in bTBI we reported previously^17^, it is plausible that both the direct deformation of astrocytes by BOP and the subsequent edema stimulated astrocytic release of ET-1 leading to the observed BBB disruption. The previously reported delayed increase in astrocytic ET_B_ receptors in the same blast injury model^87^ lends additional support to the hypothesis that dysregulated astrocytes contribute to vascular injury in bTBI. A significant finding in this study is the timing of blast-induced disruption of BBB: the increase in BBB permeability, as measured by intravital microscopy, reduction in TJ proteins, and increase in AQP4 and MMP-9 occur within 24 h after blast exposure. Although biphasic changes (early and delayed) in BBB integrity have been reported after blast injury^90^, our study demonstrates only early changes. This may be a result of differences in the blast models, as previously discussed, and the time points selected for assessment of BBB properties after blast. In the current study, although cyclic BBB disruption was not observed in our model, secondary re-opening of the barrier occurring between 3 and 14 days or 14 and 28 days post-blast cannot be ruled out. The temporal dynamics of blast-induced BBB disruption are reminiscent of the variability of temporal manifestations of blast-related alterations in cerebral microvascular reactivity and associated changes in endothelial, vascular smooth muscle, and astrocytic proteins^87^. Collectively, the findings emphasize the importance of the time dimension of blast-related vascular pathophysiology, a factor that should be carefully considered when studying potential therapeutic interventions for optimal targeting and timing of these therapies.

Another cell type that is believed to significantly contribute to the formation of BBB and maintenance of its integrity is the pericyte^91^. These cells play a pivotal role in maintaining BBB permeability by regulating the formation and function of TJs and modulating vesicular trafficking in capillary and post-capillary venular endothelial cells^92^. The effect of bTBI on pericytes is an area that remains to be fully understood. Future work will evaluate the responses of pericytes to BOP exposure and their role in bTBI.

Rapid changes in the BBB are seen as one of the first responses to bTBI, which are associated with a multitude of abnormal processes within the neurovascular unit including endothelial, astrocytic and mural cells. These early changes in the properties of the BBB and the complex mechanisms that regulate the barrier may have longer lasting secondary repercussions on the neurovascular unit with toxic effects on cerebral vasculature and parenchyma leading to neuroinflammation and neuronal degeneration^55,93^.

In summary, our findings describe size-specificity and time-dependence of alterations in the properties of the BBB and some of the associated components of the neurovascular unit in response to a single exposure to BOP. A comprehensive characterization of the time course and the extent of compromise in BBB permeability along with the associated changes in the neurovascular unit as a function of blast intensity and repetitiveness will provide an insight into understanding the complex pathophysiology of bTBI.

## Methods

### Animals

The animal study protocol was reviewed and approved by the Walter Reed Army Institute of Research/Naval Medical Research Center Institutional Animal Care and Use Committee in compliance with all applicable Federal regulations governing the protection of animals in research. The experiments reported herein were conducted in compliance with the Animal Welfare Act and per the principles set forth in the “Guide for Care and Use of Laboratory Animals,” Institute of Laboratory Animals Resources, National Research Council, National Academy Press, 2011. All experiments reported here are in compliance with ARRIVE guidelines. Animals were pair-housed with a 12 h light/dark cycle, provided ad libitum access to food and water; and acclimated for one week prior to experimental use. Male Long Evans rats (10 weeks; 300–325 g; Charles River, MD, USA) were randomly assigned to sham, 2–3 h (h), 1 day (D), 3 D, 14 D, and 28 D groups. Sham animals were euthanized 2–3 h after sham procedures. A sample (*n* = 3/group) of time matched (1 and 3 D) sham animals were evaluated to determine if exposure to sham conditions had any prolonged effects. The temporal effect of blast exposure on functional (via IVM; *n* = 6/group) and structural (via immunohistochemistry, Western blotting, enzyme-linked immunosorbent assay (ELISA), and polymerase chain reaction (PCR); *n* = 6/group) alterations in the BBB permeability were assessed in separate sets of animals.

### Blast exposure

All animals were anesthetized with 5% isoflurane for 2.5 min followed by a single exposure to the blast wave in an air driven blast simulator as described previously^94^. Briefly, the blast simulator is a long circular tube 12″ in diameter consisting of a 2.5 ft. long air compression chamber separated from a 17 ft. long expansion chamber by a polyethylene terephthalate, Mylar, membrane (Tekra, LLC. New Berlin, WI). The thickness of Mylar membrane determines the characteristics of the blast wave. The animals were placed in a restrainer in the expansion chamber with their head facing and body in line with the blast wave, while movement during the exposure was minimized by immobilizing the anesthetized animals in a restrainer. Sham animals underwent all procedures similar to other animals, except exposure to the blast wave. Blast parameters were averaged over 8 trials and are presented as mean ± standard deviation (SD). The characteristics of the positive phase (overpressure) of the primary blast wave were: peak pressure 18.9 ± 0.25 PSI, duration 10.95 ± 0.99 ms, impulse 70.26 ± 1.18 PSI.ms; and those of the negative phase (underpressure) were: peak magnitude of 1.28 ± 0.20 PSI, duration of 11.3 ± 0.91 ms, and impulse of 3.74 ± 0.47 PSI.ms.

### Surgical preparation for intravital microscopy and image analysis

Following exposure to blast or sham procedures, the animals were examined at 2–3 h, 1, 3, 14, or 28 D. On the day of the surgery, animals were weighed and anesthetized with an intraperitoneal injection of acepromazine (4 mg/kg) and ketamine (72–100 mg/kg). Buprenorphine (0.1 mg/kg) was administered subcutaneously for analgesia. Surgical plane of anesthesia was maintained subsequently with ketamine. The left femoral vein was cannulated with flexible polyethylene-50 tubing for injection of the fluorescent tracers. Two conjugated fluorescent tracers were used to assess the extent of BBB permeability: a 40 kDa tetramethylrhodamine isothiocyanate dextran (TRITC-dextran, Sigma-Aldrich, St. Louis, MO) and a 70 kDa fluorescein isothiocyanate dextran (FITC-dextran, Sigma-Aldrich, St. Louis, MO).

Pial microcirculation was accessed through a rectangular craniectomy (~ 3 × 4 mm) prepared in the right parietal bone as previously described^95–97^. The dura was resected to expose pial microvasculature. The surface of the brain was superfused with artificial cerebrospinal fluid (CSF, Harvard Apparatus, MA) to maintain brain surface electrolyte balance. A stereomicroscope equipped with a digital camera (SZ16, Olympus, Japan) was used for imaging the pial microcirculation. A total magnification of 40X and a resolution of 0.8063 pixel/micrometer for both X and Y dimensions were used for image acquisition. Following a 10-min stabilization period after cranial window implantation, an intravenous injection of TRITC-dextran (25 mg/kg/1.5 mL PBS) was administered and the pial microcirculation was imaged for 5 min starting immediately after injection. A 30-min wash-out period was allowed before the injection of the larger tracer, FITC-dextran (25 mg/kg.1.5 mL PBS), which was also imaged in the pial microcirculation for 5 min. Images were analyzed using Image J software (National Institutes of Health, Bethesda, MD) as follows. The para-arteriolar and para-venular sites with extravasation of the tracers were identified as regions of interest (ROI). The fluorescence intensity at these ROIs was estimated for images at one-minute intervals of the 5 min of imaging. The ROIs were reproduced (same location and size) in all images obtained from the animal to track changes in tracer extravasation from microvessels. A permeability index (PI) was calculated from the images obtained at one-minute intervals after tracer injection using the following formula:

$$\mathrm{Permeability index }(\mathrm{PI}) = \mathrm{\frac{ Intensity\left({t}_{n}\right)-Intensity({t}_{0})}{Intensity({t}_{0})},}$$

where, *n* = 1, 2, 3, 4, 5 min and t_0_ is the baseline immediately after tracer administration. For each animal, an average PI over 5 min was derived which was used to calculate the mean (and SD) of the index within a group. In total, 30 ROIs per group (6/animal) were used for calculation of PI.

### Immunohistochemistry, immunofluorescence, and fluorescence quantification

To examine vascular changes in endothelial TJ proteins associated with BBB disruption in the cortex after exposure to BOP, the expression of TJ proteins, ZO-1, claudin-5, and occludin in cortical vessels was examined using immunohistochemistry. The localization of the TJ proteins to the vascular wall was assessed by combining the TJ protein antibodies with antibodies against the contractile smooth muscle marker smoothelin (SMTH). In addition, changes in the water channel protein, aquaporin 4 (AQP-4) were also examined to understand the effects of blast exposure on water transport across the BBB. The animals exposed to sham or blast procedures were euthanized with sodium pentobarbital (1 ml/kg, Euthasol, Vibrac AH Inc., Fort Worth, TX) at specified study endpoints after the exposure and the brains were harvested and immediately embedded in optimal cutting temperature compound (Tissue-Tek OCT compound, Sakura Finetek Europe B.V) over dry ice. Cryostat sections (5 μm) from 0.8 mm anterior to- and 4.8 mm posterior to bregma were processed for immunohistochemistry using standard methods. Briefly, tissue sections were washed in PBS for 15 min and blocked in 2% bovine serum albumin in PBS for 30 min at room temperature. The sections were then incubated with rabbit anti-SMTH (1:2000, Invitrogen, Carlsbad, CA), mouse anti-ZO-1(1:500, Invitrogen), mouse anti-occludin (1:500, Invitrogen), mouse anti-claudin-5 (1:500, Invitrogen), and rabbit anti AQP-4 (1:1000, Santa Cruz Biotechnologies, Dallas, TX) overnight at 4 °C. After washes in PBS, the sections were incubated with the appropriate Cy2- or Cy3- conjugated secondary antibodies (1:500; Jackson ImmunoResearch, West Grove, PA) for 2 h at room temperature or overnight at 4 °C. Following washing in PBS, sections were dehydrated with alcohol, cleared with xylene, mounted on slides with Permount mounting medium (Electron Microscopy Sciences, Hatfield, PA) and coverslipped. The co-localization of TJ proteins and SMTH was visualized by confocal laser scanning microscopy (Fluoview FV1200, Olympus). The immunofluorescence of TJ proteins was examined with an Olympus AX80 (Olympus) and images were captured with a DP72 camera (Olympus) using the software cellSens Dimension (Olympus). Immunofluorescence intensity of TJ proteins, SMTH, and AQP-4 was quantified by Image Pro (Media Cybernetics Inc., Rockville, MD). For each animal 4–6 sections were selected, and 3 images with visible vessels per tissue section were taken. A total of 80–90 vessels in each group were analyzed. Image background subtraction was achieved by adjusting the contrast and converting the image to black and white, with white representing staining of the target proteins. The average and SD values of immunofluorescence intensity for each group was determined.

### Western blots

The animals from sham or blast conditions were euthanized at specified study endpoints post-blast. Fresh brain tissue was collected, flash frozen in liquid nitrogen, and stored at − 80 °C until further processing. Frontoparietal cortical tissue lysates were prepared in a buffer containing 150 mM NaCl, 50 mM Tris–HCl, 0.25% deoxycholate, 1 mM EGTA, 1 mM NaF, 1 mM Na_3_VO_4_, and a cocktail of proteinase inhibitors. Immunoblots were prepared as previously described^98^. Briefly, protein samples from individual rats (25 µg/rat/well) were loaded onto 4–12% Tris-bis gels and, after electrophoresis, transferred to polyvinylidene fluoride (PVDF) membranes. The membranes were incubated with primary antibodies against claudin-5 (1:1000; Thermo Fisher Scientific, Waltham, MA), occludin (1:1000; Thermo Fisher Scientific), or ZO-1 (1:2000; Thermo Fisher Scientific) at 4 °C overnight. After several washes, the membranes were incubated with the appropriate HRP-conjugated anti-rabbit or anti-mouse secondary antibodies (1:5000; Cell Signaling Technologies, Danvers, MA) at room temperature. Each membrane was probed for the housekeeping protein β-actin using a mouse monoclonal antibody (1:10,000; Sigma, St. Louis, MO). Chemiluminescence was detected using an enhanced chemiluminescence reagent (SuperSignal West Femto, Thermo Fisher Scientific) and imaged in LAS-3000 Luminescent Image Analyzer (Fujifilm). Semi-quantitative assessment of immunoblot band optical density was acquired using Image J software. Band density for each sample studied was normalized to that of β-actin for the same sample and then compared to sham animals. All data is presented as mean ± SD.

### Enzyme-linked immunosorbent assay (ELISA)

To examine changes in MMP-9 protein levels, blood and brain tissue were collected from anesthetized rats. Plasma was isolated from EDTA-anticoagulated blood samples by centrifugation at 1000×*g* for 15 min at 4 °C and stored at -80 °C until further analyses. The frontoparietal cortical tissue was rinsed in cold PBS (0.01 M, pH 7.4) to remove excess blood and immediately frozen in liquid nitrogen and stored at − 80 °C. The tissue was homogenized in 0.01 M PBS supplemented with protease and phosphatas e inhibitors. Tissue lysates were centrifuged at 12,000×*g* for 10 min at 4 °C. Supernatants were collected and protein concentrations were measured using a BCA Protein Assay kit according to the manufacturer’s recommendations (Thermo Fisher Scientific, Waltham, MA). MMP-9 levels were measured with quantitative sandwich ELISA techniques with 100 µl plasma or 100 µg/100 µl tissue protein according to the manufacturer’s instructions (MyBioSource, San Diego, CA). The concentration of MMP-9 in plasma is expressed as ng/ml and the levels of MMP-9 in the brain tissue as ng/mg of protein.

### Real-time polymerase chain reaction (RT-PCR)

MMP-9 gene expression was evaluated by RT-PCR. Frontoparietal cortical tissue from harvested brains was stored in RNAlater overnight at 4 °C before transferring to − 80 °C. Total RNA was extracted by Trizol (Invitrogen, Carlsbad, CA) following manufacturer’s instructions. Qualities and quantities of RNA were checked by Agilent RNA 6000 Nano Kit (Agilent Technologies, Santa Clara, CA) and Nanodrop (Thermo Fisher Scientific, Wilmington, DE). cDNA was prepared by Reverse-Transcriptase of total RNA by Qiagen RT^2^ First Strand Kit (Qiagen Science, Germantown, MD). Amplification of cDNA occurred with RT^2^ SYBR Green ROX Mastermix at 95 °C for 10 min followed by 40 cycles for 15 s at 95 °C and 1 min at 60 °C (ABI Prism 7900 H T Sequence Detection System, Applied Biosystems Inc., Foster City, CA). Primers for MMP-9 and β-actin were Qiagen’s RT^2^ qPCR Primer products. The relative expression of mRNA was normalized to the amount of β-actin in the same cDNA sample using the relative quantification ΔΔCt method described by the manufacturer. Results from the sham group were used as calibrators to calculate relative quantitative MMP-9 expression.

### Data and statistics

Investigators were not blinded to the treatment of animals in various groups due to multiplicity of roles performed by each investigator. However methodological precautions were incorporated in the study to minimize any potential bias. Duplicate assessment of outcomes from image analyses were conducted to minimize subjectivity and ensure reliability of the results. All data are presented as mean ± SD. Shapiro-Wilks methods were used to test that the assumptions of normality were not violated; ANOVAs were used if parametric methods were acceptable, while non-parametric methods, such as Kruskal–Wallis or other non-parametric equivalents, were used when the assumptions could not be met. The intergroup differences were determined by one-way ANOVA followed by Dunnett's multiple comparisons test, or by a Kruskal–Wallis test followed by Dunn’s multiple comparisons test, with Bonferroni-adjusted *p* ≤ 0.05 considered to be significant; if parametric methods were used, data is reported as means and SD, while for non-parametric methods the data is reported using the median and interquartile range (25th to 75th percent interval). The effect sizes, with corresponding 95% confidence intervals, are reported as partial *η*^2^. The analyses were done using GraphPad Prism software (GraphPad Software Inc., San Diego, CA).

### Disclaimer

 The views expressed in this manuscript are those of the author and do not necessarily reflect the official policy or position of the Department of the Navy, Department of Defense, nor the U.S. Government. This work was supported/funded by work unit number 603115HP.3520.001.A1411. The study protocol was reviewed and approved by the Walter Reed Army Institute of Research/Naval Medical Research Center Institutional Animal Care and Use Committee in compliance with all applicable Federal regulations governing the protection of animals in research. The experiments reported herein were conducted in compliance with the Animal Welfare Act and per the principles set forth in the "Guide for Care and Use of Laboratory Animals," Institute of Laboratory Animals Resources, National Research Council, National Academy Press, 2011. Some of the authors are military Service members [or employees of the U.S. Government]. This work was prepared as part of their official duties. Title 17, U.S.C., §105 provides that copyright protection under this title is not available for any work of the U.S. Government. Title 17, U.S.C., §101 defines a U.S. Government work as a work prepared by a military Service member or employee of the U.S. Government as part of that person’s official duties.

## Supplementary Information

Supplementary Information.
