# Supplementary material for: Blast-induced temporal alterations in blood–brain barrier properties in a rodent model
Source: Sci Rep. 2021 Mar 15;11:5906. doi: 10.1038/s41598-021-84730-8 (PMC7971015; doi:10.1038/s41598-021-84730-8)
Supplement: Supplementary file 1 — Supplementary Information. [file 41598_2021_84730_MOESM1_ESM.pdf]

Blast-induced Temporal Alterations in Blood-Brain Barrier Properties in a Rodent Model

Usmah Kawoos[1,2]*, Rania Abutarboush[1,2], Ming Gu[1,2], Ye Chen[1,2], Jonathan K. Statz[1,2], Samantha Y. Goodrich[1,2], Stephen T. Ahlers[1]

[1]Neurotrauma Department, Naval Medical Research Center, Silver Spring, Maryland

[2]The Henry M. Jackson Foundation for the Advancement of Military Medicine Inc., Bethesda, Maryland

*Corresponding author: Usmah Kawoos,

Neurotrauma Department,

Naval Medical Research Center,

503 Robert Grant Ave.,

Silver Spring, Maryland 20910

Phone: 301-319-2058; Email: Usmah.kawoos.ctr@mail.mil

Running headline: Longitudinal effects of blast on blood-brain barrier

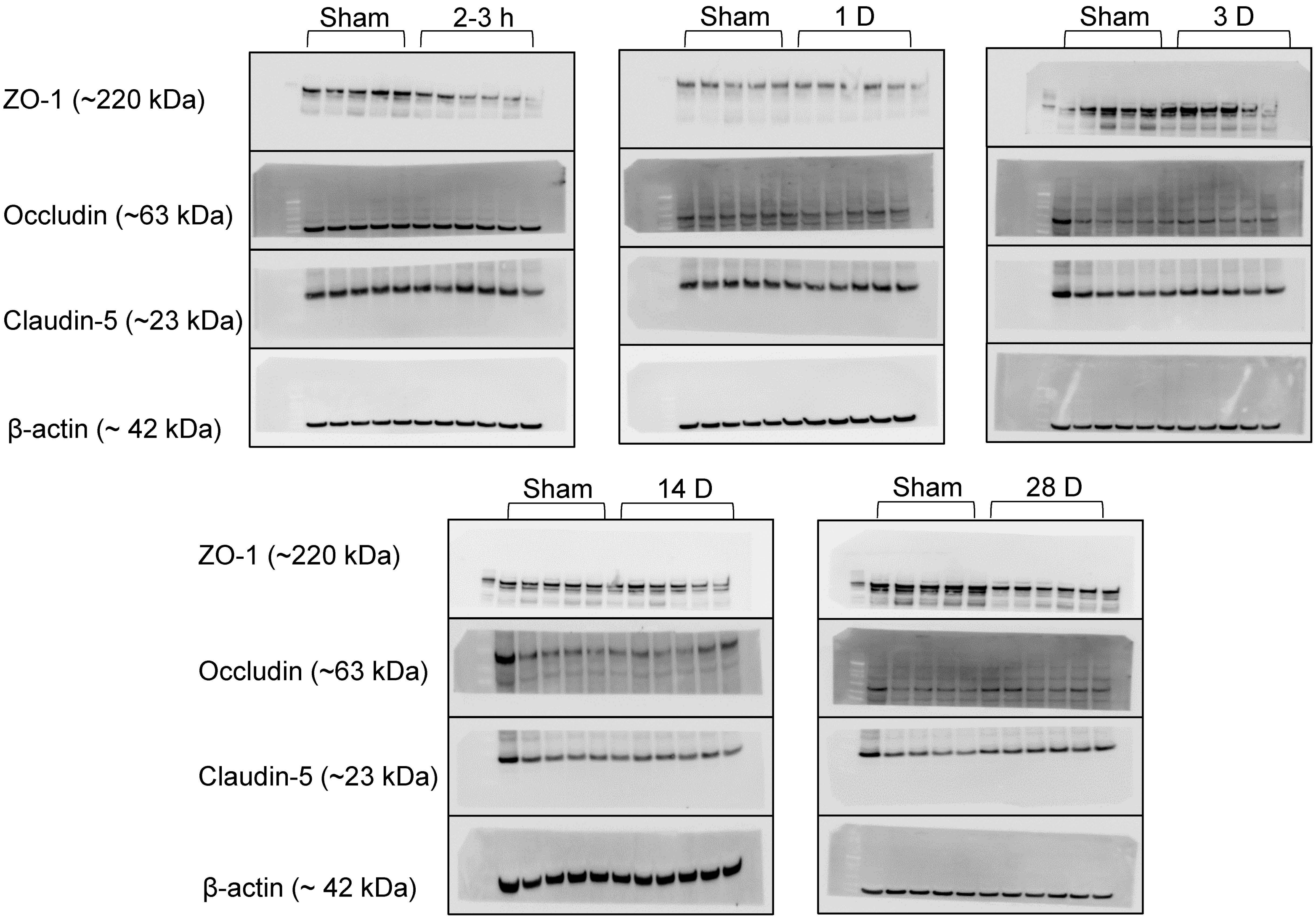

Supplementary file: Comparison of Western blot bands for tight junction proteins (ZO-1 (zona occluden-1), occludin, and claudin-5) between sham and post-blast groups at 2-3 h (hours), 1, 3, 14, and 28 D (days). The TJ proteins for a specified post-blast time point were probed on a single membrane. Since the molecular weights of the proteins of interest are well separated each membrane was cut horizontally at 80 kDa and 30 kDa. The part of the membrane with proteins >80 kDa was probed for ZO-1, while the membrane with proteins 30-80 kDa was probed for occludin. This section of the membrane was incubated in stripping buffer and then re-probed for the house-keeping protein β-actin. The part of the membrane with <30kDa protein was probed for claudin-5.
